# The Eclampsia Escape Room: An Innovative Educational Experience for the OB/GYN Clerkship

**DOI:** 10.15766/mep_2374-8265.11629

**Published:** 2026-08-28

**Authors:** Shreya Venkat, Gabriela Guerra, Carolyn Davis, Amanda Slater, Erick Garcia, Heather Wolfe

**Affiliations:** 1 Medical Student, University of Virginia Inova Campus; 2 Resident, Department of OB/GYN, Inova Health System; 3 Associate Professor, University of Virginia Inova Campus; 4 Clerkship Coordinator, University of Virginia Inova Campus; 5 Senior Simulation Technician Coordinator, Inova Center of Advanced Medical Simulation; 6 Assistant Professor, University of Virginia Inova Campus and Inova Health System

**Keywords:** OB/GYN, Obstetrics and Gynecology, Games, Gamification, Pre-eclampsia, Eclampsia

## Abstract

**Introduction:**

Escape rooms are a popular form of entertainment in which participants collaborate to solve puzzles to achieve a goal, often “escaping” a scenario. In educational settings, escape rooms are grounded in theories of gamification and experiential learning, with evidence supporting knowledge acquisition and learner satisfaction in health professions education. Despite increasing use in medical education, escape rooms remain underutilized in undergraduate obstetrics and gynecology (OB/GYN) training. Hypertension in pregnancy and eclampsia are critical components of undergraduate medical education and good subjects with which to use this immersive educational experience.

**Methods:**

Third-year medical students on the OB/GYN clerkship participated in a 60-minute lecture-based didactic, followed by an eclampsia-themed escape room of approximately 20 minutes. Learning objectives were reviewed before both activities. The escape room scenario involved a labor and delivery patient who acutely “does not feel well.” Students were oriented to expectations and ground rules before working through a series of puzzles leading to a final passphrase. A structured debrief followed, during which each puzzle was reviewed and explicitly mapped to the learning objectives.

**Results:**

Fifty-eight medical students participated in the escape room, and 15 students (25.9%) completed the postactivity survey. All respondents reported being satisfied or very satisfied with the escape room as part of their educational experience. All participants indicated that the activity added value or significant value and that learning objectives were met.

**Discussion:**

This escape room represents a novel educational strategy for OB/GYN clerkship students and demonstrates perceived educational value and achievement of learning objectives.

## Educational Objectives

By the end of this activity, learners will be able to:

1. Describe how normal pregnancy physiology changes and how the pathophysiology of pre-eclampsia differs.
2. Diagnose and distinguish gestational hypertension, pre-eclampsia, pre-eclampsia with severe features, chronic hypertension, and chronic hypertension with superimposed pre-eclampsia.
3. Manage severe hypertension in pregnancy with appropriate medications and algorithms.
4. Order and interpret labs for the evaluation of pre-eclampsia.
5. Manage a seizure in pregnancy including use of magnesium sulfate and appropriate precautions.
6. Discuss potential sequelae of pre-eclampsia and future considerations for prevention.

## Introduction

Hypertensive disorders of pregnancy, including gestational hypertension, pre-eclampsia, and eclampsia, remain major contributors to maternal morbidity and mortality and are critical components of core curricular content in undergraduate medical education.^1,2^ It is important for clerkship medical students to be adequately trained in this subject for many reasons, including medical licensing exams and future clinical practice. Many current instructional approaches for this subject matter include a lecture with or without a simulation, though some facilitators note a declining level of learner engagement with reports of “simulation fatigue,” where emotional load, cognitive overload, or repetitive exposure diminishes the effectiveness of simulation-based education.^3,4^

Educational escape rooms have recently emerged as novel, gamified instructional strategies shown to increase motivation, collaboration, and active problem solving in medical education.^5-7^ While escape rooms have been applied across various health professions domains, few published resources describe intentional alignment with clinical learning objectives, and fewer still address their use in teaching pre-eclampsia and eclampsia specifically. This represents a gap and an opportunity to introduce a theoretically grounded, engaging adjunct to traditional approaches. Our project further offers both high- and low-resource implementation pathways, strengthening its reproducibility across institutions with varying simulation capabilities.

This activity is inspired by a desire to create a unique experience that encourages increased involvement, motivation, and knowledge retention. This activity is informed by multiple educational frameworks. Game-based learning utilizes emotions in the learning process and increases “engagement, motivation, and efficiency within the context of the learning environment.”^8^ Game-based learning refers to the use of games or game-like elements to engage learners and enhance the educational experience, and has been noted to particularly appeal to Millennials and Generation Z, who compose a majority of current medical students.^8^ Game-based learning incorporates principles of constructivist learning, which emphasizes knowledge building through active, social interaction, as well as experiential learning, which prioritizes memorable, hands-on experiences over passive memorization.^9,10^ Cognitivism also supports this instructional approach: it posits that learners form new memories by integrating new information into existing knowledge structures and benefit from actively directing their own learning. Offering multiple modalities—lecture followed by an interactive escape room—provides varied opportunities to process, organize, and reinforce content, strengthening cognitive pathways and promoting deeper retention. Nontechnical skills such as teamwork and communication have also been hypothesized to improve through participation in educational escape rooms.^8,10^ These educational frameworks, combined with the need for an interactive and memorable approach to high-stakes obstetric emergencies, provided the rationale for developing a structured escape room activity aligned with core clerkship learning objectives.

## Methods

### Development

We implemented this instructional activity within the obstetrics and gynecology (OB/GYN) clerkship at the University of Virginia Inova Campus in 2025. Third-year medical students rotating on the OB/GYN clerkship served as the target learners. This learning activity occurred after a lecture on hypertensive disorders of pregnancy to create a unique experience that encouraged increased involvement, motivation, and knowledge retention. We piloted the escape room prior to implementation in the clerkship with OB/GYN residents and senior students at our institution and incorporated feedback from these sessions and from other facilitators into the final product. The Institutional Review Board at Inova approved this study as exempt (INOVA-2024-341).

### Equipment/Environment

We did not assign specific prereading prior to this activity, which consisted of a 1-hour didactic component and a 20-minute escape room component. We conducted the didactic component in a classroom with a projector screen to review a PowerPoint on hypertensive disorders in pregnancy (Appendix A). We conducted the escape room in a labor and delivery simulation room with a high-fidelity childbirth patient simulator in a low lithotomy position. The room had 2 table spaces on which the learners solve puzzles, as well as a TV monitor used to display a code for the final pass phrase. Due to resource availability, it is possible to perform this activity without a manikin and using printable puzzles. The choice of experience should be related to resource availability.

The list and location of items needed for the escape room can be viewed in the room layout document and are broken down into required and optional equipment for high- and low-resource options (Appendix B).

### Personnel

Facilitators included faculty clinicians with content expertise in the diagnosis and management of hypertensive disorders of pregnancy; no specialized simulation or game-based training is necessary. A simulation specialist also attended our encounter to run a simulated eclamptic seizure during the escape room, as well as help with equipment troubleshooting. This is an optional level of the escape room for high-resource settings and is not necessary for basic implementation. A member of the study team provided facilitators with a copy of the escape room appendices and oriented them to all parts of the escape room prior to the activity sessions (Appendix C). The orientation lasted no more than 15 minutes and included a review of the learning objectives, each of the puzzle steps, common areas of difficulty, ways to prompt or support learners encountering difficulties, and the debrief steps. We gave faculty the opportunity to ask questions and receive clarification prior to the activity. With this preparation, facilitators were prepared for the event without the need for any formal simulation training.

### Implementation

All students attended an approximately 1-hour in-person didactic session covering the learning objectives of the hypertension in pregnancy module, including the Eclampsia Escape Room (see Appendix A). We gave students time to ask questions during the didactic session. We then divided the learners into groups of 3 to 6 students per group. We then gave students a prebrief and instructions about the escape room (Appendix D).

The students were then able to begin the Eclampsia Escape Room. We designed each puzzle so that successful completion unlocked the next step, culminating in a final “win” once all learning objectives had been achieved. Faculty would observe from the simulation control room and could use a microphone to provide hints if students required it. The facilitators used Appendix C, containing a detailed description of the ideal progression of the escape room, to guide students as needed. The first step required the students to recognize the diagnostic criteria for pre-eclampsia. The second step required the students to select appropriate medications to treat acute severe range blood pressures. The third step required students to select appropriate labs for further evaluation of pre-eclampsia, including assessment of disease severity. When students started on the fourth step, the simulation technician would cause the manikin to seize, which was an engaging part of the experience but is not a required component of this experience, though it can be considered in high-resource settings. The fourth step required the students to identify ways to help manage the seizure, including positioning the patient and the bed, calling for help, and initiating other interventions recommended in this setting. The final step of the escape room activity was solving a crossword puzzle and using a decoder projected on the TV (Appendix E) to spell out “We love OB/GYN,” at which point the TV screen displayed a message that read: “You win!” At this point, the facilitators entered the room to debrief the activity using Appendix F. Appendix B includes all required and optional items, including considerations for high- and low-resource settings and some real-world example materials.

### Debriefing

Immediately following completion of the activity, each group participated in a 10-minute debrief of the learning activity, facilitated by the observing faculty members. Debriefing focused on reviewing the diagnostic criteria for pre-eclampsia, medications used to treat severe-range blood pressures, laboratory tests useful in the initial workup of pre-eclampsia, and actions necessary in the management of an eclamptic seizure. If groups had difficulty in specific parts of the escape room or required hints, we discussed what contributed to these challenges and addressed any knowledge gaps. We also reviewed teamwork and communication strategies during the debrief. We used Appendix F to facilitate the debrief.

We recommend conducting the debrief immediately after the escape room concludes, as the learners are still invested in the game and eager to learn from their mistakes. We conducted the debrief in the same room so that the puzzles and components could be held and reviewed as we discussed each step.

### Assessment

We gave students a link following the escape room to a postintervention feedback survey (Appendix G). We created this 6-question survey including multiple choice, yes/no, and open-ended questions to gain more information about student perceptions and the experience of participating in the escape room. The survey was not formally pilot tested prior to administration. This survey examined students’ experiences and satisfaction with using the escape room as an adjunct to their education. Participation in the postintervention survey was optional.

## Results

The escape room experience has been implemented 12 times. Facilitators have included 4 OB/GYN faculty members, 1 OB/GYN resident, and 1 senior medical student. A total of 58 students participated in the Eclampsia Escape Room, and 15 (25.9%) completed the optional postactivity survey. All respondents agreed that participation in medical education research is an important contributor to improving clinical teaching and patient care.

Overall satisfaction with the Eclampsia Escape Room was high, with 80% (*n* = 12) of participants reporting that they were “very satisfied” and 20% (*n* = 3) reporting that they were “satisfied.” Participants also reported that the activity contributed meaningfully to learning: 60% (*n* = 9) indicated that the session added “significant value” and the remaining 40% (*n* = 6) that participation in the Eclampsia Escape Room added “value” to the overall educational experience. All participants indicated that the Eclampsia Escape Room met the intended learning objectives.

Qualitative responses were largely positive and reflected recurrent descriptions of the activity as engaging, enjoyable, and high yield. When asked why the activity met the proposed learning objectives, commonly referenced themes included engagement, reinforcement of content, high-yield format, fun, effectiveness, and interactivity. Participants frequently characterized the activity as clear, relevant, and well structured. When describing what worked well, the most commonly cited themes included clarity, relevance to content, and overall enjoyment or fun. Several respondents also highlighted features that encouraged collaboration, problem solving, and recall of material from the preceding lecture (Figure).

**Figure.**
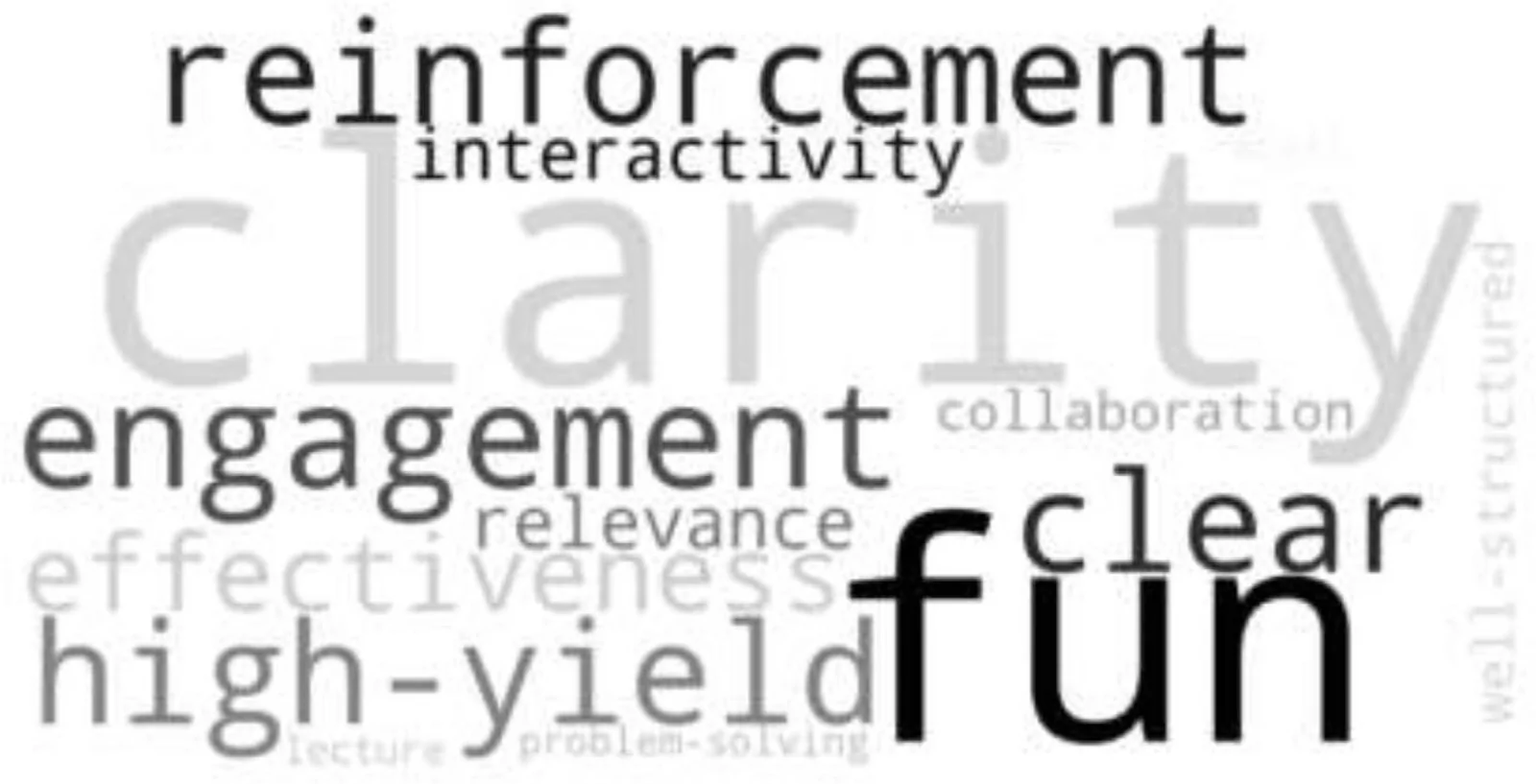
Word cloud representing themes identified from qualitative survey responses. Word size corresponds to the frequency with which each theme was referenced by participants. The most commonly cited themes included clarity (33%), fun (27%), engagement (20%), reinforcement of content (20%), and high-yield format (20%), reflecting overall positive perceptions of the educational activity.

Some participants identified elements that were less effective or potentially confusing. Recurring concerns included difficulty with the combination lock and uncertainty about manipulating or moving the manikin. Suggestions for improvement focused on increasing the number of simulation elements, providing more explicit guidance, and ensuring that puzzle components and equipment function reliably. The respondents provided clear, specific examples of elements that were successful and those that could be improved throughout the Eclampsia Escape Room, demonstrating the value of completing the postintervention feedback survey as a guide for continued improvement in the project.

## Discussion

This educational experience was created to supplement a lecture and can be easily adapted by other institutions seeking an engaging, novel way to interact with the material. While our original Eclampsia Escape Room utilized a simulation room, seizing manikin, and hand-constructed puzzle pieces, these additional time and resource-intensive components are not necessary, and it is possible to have this experience in a low-resource setting as specified in detail in Appendix B to maximize generalizability.

This experience can be adapted to any number or makeup of learners. For example, the experience could be implemented in a setting with a larger number of students by breaking into tables of 5-10 students each and having them work through each step at their own table, with senior students or residents supervising. There are many ways that this experience could easily be adapted to the unique circumstances and resources available at different institutions.

Overall, the quantitative and qualitative data demonstrate that the Eclampsia Escape Room was perceived as an educationally valuable, engaging, and high-yield learning modality that effectively reinforced the targeted clinical objectives, though it is acknowledged that we had a low response rate. Possible causes for limited responses could be the lack of a direct incentive for survey participation, robust clinical and academic demands making optional tasks a low priority, and survey overload from frequent requests to complete evaluations throughout the medical clerkships. All possible factors could be taken into consideration for further studies to increase response rate. Future investigation can include knowledge assessment or real-world application of skills or behaviors. The opt-in survey approach, specifically, carries known limitations such as overrepresentation of strongly held views and selection bias, and future research directions could include objective assessments such as pre- and postknowledge quizzes or objective performance measures to supplement these findings. Future iterations of this experience can incorporate pre/postconfidence levels, knowledge assessments, and objective performance measures for a stronger assessment of educational value.

## Appendices


Hypertension in Pregnancy.pptxRoom Layout and Materials.pptxCase Template and Facilitator Guide.docxOrientation for Students.docxCrossword and Passphrase.pptxDebrief Tool.docxFeedback Survey.docx
*All appendices are peer reviewed as integral parts of the Original Publication.*
