# Supplementary material for: The Eclampsia Escape Room: An Innovative Educational Experience for the OB/GYN Clerkship
Source: MedEdPORTAL. 2026 Aug 28;22:11629. doi: 10.15766/mep_2374-8265.11629 (PMC13522248; doi:10.15766/mep_2374-8265.11629)
Supplement: Supplementary file 1 [file mep_2374-8265.11629-s001.zip › C. Case Template and Facilitator Guide.docx]

**Appendix C:** This serves as faculty preparation and orientation document to review all important steps as well as a detailed description of the escape room itself and all components and steps.

| Appendix C: Escape Room Guide  TITLE: **The Eclampsia Escape Room: An Innovative Educational Experience for the OB/GYN Clerkship**  AUTHORS: Shreya Venkat, Gabriela Guerra, Carolyn Davis, Amanda Slater, Erick Gracia, Heather Wolfe  LEARNER AUDIENCE: Third Year / OB/GYN Clerkship Medical Students | |
| --- | --- |
| Patient Info: 40 yo G2P1001 at 40w5d  PHYSICAL SETTING: Inpatient labor and delivery simulation room (high resource) or quiet space with table and chairs (low resource) | |
| Brief narrative description of case | *Background: You are the intern on L&D days. This patient is Emily, a 40 yo G2P1001 at 40w5d who has just started pushing with the nurse. The nurse wants you to come see the patient as she “just does not feel well”.*  *-------------------*  *Instructions (high resource):* *This is NOT a simulation (i.e. you are not expected to talk with the patient or actually perform procedures or give medications) but you should explore and interact with the environment, turn things over, etc, in order to find clues and work together to solve a series of puzzles in order to figure out the final pass phrase. This will “win” the escape room!*  *Ground rules:*  *1) Don’t force anything open if you do not yet have the correct clue or answer to open a box*  *2) Don’t write on anything but paper*  *3) Stay within the curtained area*  *4) You can always leave if you need to*  *--------------------*  *Instructions (low resource): This is a series of puzzles that you will solve in order to find clues and work together to figure out the final pass phrase. This will “win” the escape room!*  *Ground rules:*  *1)Don’t force anything open – you will receive permission from the facilitator to open envelopes once you have achieved the current puzzle step*  *2)You will be provided with all materials to complete the puzzles, you do not need to explore the room*  *3)You can always leave the room if you need to (you are not locked in!)* |
| Learning Objectives | *By the end of this activity, learners will be able to:*  *1. Describe how normal pregnancy physiology changes and how the pathophysiology of pre-eclampsia differs.*  *2. Diagnose and distinguish gestational hypertension, pre-eclampsia, pre-eclampsia with severe features, chronic hypertension, and chronic hypertension with superimposed pre-eclampsia.*  *3. Manage severe hypertension in pregnancy with appropriate medications and algorithms.*  *4. Order and interpret labs for the evaluation of pre-eclampsia.*  *5. Manage a seizure in pregnancy including use of magnesium sulfate and appropriate precautions.*  *6. Discuss potential sequelae of pre-eclampsia and future considerations for prevention.* |
| Key Puzzle Steps | *1) Understand the diagnostic criteria for pre-eclampsia and apply it to a specific patient to obtain the code to open the first box (high resource) or permission to open the large manila envelope (low resource)*  *- Match “headache” and “elevated blood pressure” puzzle pieces*  *2) Recognize the need for urgent treatment of severe range blood pressure and use the correct medications in management to obtain the code to open the zipper pouch (high resource) or permission to open the small envelope (low resource)*  *- Correctly identify IV hydralazine, IV labetalol, PO nifedipine IR*  *3) Order appropriate labs to further evaluate for pre-eclampsia to complete the puzzle*  *- Accurately include CBC, creatinine, AST/ALT, urine protein to creatinine ratio, LDH, uric acid*  *4) Manage a seizure including calling for help, patient positioning, airway management, magnesium sulfate administration, and other room and safety factors to find all crossword clues around the room (high resource) or be given all crossword clues (low resource)*  *- Find the clues labeling different seizure management items (high resource) or ask for them (low resource)*  *5) Apply knowledge about physiology and other considerations for pre-eclampsia management to complete the final crossword*  *- Complete the crossword clues correctly* |
| Learner Preparation or Prework | Learners will attend a one hour large group didactic covering the material pertaining to the learning objectives as well as a prebriefing describing the scenario and the rules prior to beginning the activity. There is no specified pre-reading before this experience. |

| Initial Presentation | |
| --- | --- |
| Initial Vital Signs | 165/85, 108, 16, 98% on RA  Fetal heart rate tracing: baseline 165, moderate variability, no accelerations, no decelerations |
| Overall Setting and Appearance | *High resource setting: the labor and delivery simulation room has a projected screen with blank crossword and cipher. There is a manikin positioned in low lithotomy with the bed broken down for delivery. There are cards taped to various locations in the room (listed in Appendix B). In the center of the room is a locked box and the first clue “What diagnostic criteria does your patient have for pre-eclampsia?”*  *-------------------------------------*  *Low resource setting: the group gathers around a table together with the facilitator. There is a large manila envelope in the center of the table and the first clue “What diagnostic criteria does your patient have for pre-eclampsia?”* |
| Standardized Participants (and Their Roles in the Room at Case Start) | *There are no additional participants in the room.*  *High resource: the facilitator watches from outside (control room or similar) or may watch from the side, does not participate unless the learner is struggling and needs a clue.*  *Low resource: the facilitator sits at the table with the group or just off to the side. The facilitator is active when the group believes they have solved the clue to give them permission to open the next envelope (steps 1 and 2) or give the clues (step 3).* |
| HPI / history / examination of manikin | *There are no questions asked of the manikin in this escape room scenario. If the student attempts to interact with the manikin by asking questions in the high resource scenario the facilitator will remind the student that this is not a simulation and they should instead interact with their environment in a way to solve the series of puzzles and redirect them to the clue that they are on.* |

Steps/Flow of Escape Room

***High resource:***

- *The group of learners is oriented outside the LDR simulation room. They are given 2-3 of the initial puzzle pieces each and told to enter the simulation room, the timer is started.*
- *When the learners enter the room they immediately see a locked box in front of them as well as the clue “what diagnostic criteria does your patient have for pre-eclampsia?” They recognize that the starting puzzle pieces have patient factors including history, vital signs, and exam findings. They correctly recognize that the severe range blood pressure and the presence of a headache are both part of the diagnostic criteria for pre-eclampsia and put those puzzle pieces together. The other patient factors are distractors. On the back of the puzzle is the code that unlocks the first box.*
- *Within the first box is another locked smaller box as well as many vials and bottles of medication. There is also a clue on the inside lid of the larger box that asks “what medications are used to treat acute severe-range blood pressure?” The students correctly sort through the options and pick IV labetalol, IV hydralazine, and oral nifedipine. The other medications are distractors. As there is no currently-established correct order of use of these medications it will be via trial and error to determine the code based on the three numbers or the clue can be altered to tell the team to place in alphabetical order for ease of use. This code unlocks the smaller box.*
- *Within this box are many different puzzle pieces, only 6 of which go together. Within the smaller box there are also black light flashlights that are used for the next step. The inside lid of the smaller box contains the next clue that asks “what labs do we order when evaluating for pre-eclampsia?” The students correctly recognize the labs as cbc, creatinine, ast / alt, urine protein to creatinine ratio, ldh, and uric acid and put this puzzle together. The other pieces that do not connect are distractors. On the back of this puzzle is the phrase “manage this seizure! Hint: remember the whole patient and room.”*
- *The manikin seizes at this point. The students must interact with the room to find all of the cards that serve as ways to keep the patient safe during the seizure, call for help, and manage the situation. The seizure stops when all cards are located. The cards are on the following locations: call bell / button, broken down foot of bed, side rail of bed that is down, cpr button or lever to lay bed flat, oxygen facemask, suction tubing, magnesium sulfate bag, back of patient manikin. In the optional setting the black light is used to reveal the crossword clues written on the cards. The cards can also have the clues printed or written on them without the use of a blacklight pen.*
- *The students complete the crossword and then use the cipher to determine the final passphrase as “We love OB/GYN!” When the facilitator hears the final phrase stated, the projected screen is then manually changed by the facilitator to say “You win!”*
- *The facilitator then enters the room to debrief each step and connect to learning objectives.*

***Low resource:***

- *The group of learners is oriented while sitting around a table. They are given 2-3 of the initial puzzle pieces each and told to begin the escape room, the timer is started.*
- *The learners immediately see a large manilla envelope in front of them as well as the clue “what diagnostic criteria does your patient have for pre-eclampsia?” They recognize that the starting puzzle pieces have patient factors including history, vital signs, and exam findings. They correctly recognize that the severe range blood pressure and the presence of a headache are both part of the diagnostic criteria for pre-eclampsia and put those puzzle pieces together. The other patient factors are distractors. The facilitator instructs the group that they have “unlocked” and can open the large manila envelope.*
- *Within the large manila envelope is another small envelope as well as many printed images of vials and bottles of medication. There is also a clue inside the large manila envelope that asks “what medications are used to treat acute severe-range blood pressure?” The students correctly sort through the options and pick IV labetalol, IV hydralazine, and oral nifedipine. The other medications are distractors. The facilitator will then instruct the group that they have “unlocked” and can open the small envelope.*
- *Within this envelope there are many different puzzle pieces, 6 of which go together. There is also a clue that asks “what labs do we order when evaluating for pre-eclampsia?” The students correctly recognize the labs as cbc, creatinine, ast / alt, urine protein to creatinine ratio, ldh, and uric acid and put this puzzle together. The other pieces that do not connect are distractors. On the back of this puzzle is the phrase “manage this seizure! Hint: remember the whole patient and room.”*
- *The group must then discuss with the facilitator all of the important components to ask for, check, and initiate when managing a seizure. The items that the team must discuss are: turn patient to the side, obtain IV access, give magnesium sulfate, apply supplemental oxygen, suction airway as needed, put bed together if broken down for delivery, make sure all handrails are up, put head of bed flat, and call for help. The students are given crossword clues that correspond to each correctly identified seizure management pearl.*
- *They solve the crossword clues to complete the crossword and use the cipher to determine the final passphrase as “we love OB/GYN!” When the facilitator hears the final phrase, they congratulate the team on solving the series of puzzles and the encounter has completed.*
- *The facilitator then debriefs each step and connects to learning objectives.*

| **Step 1:** each student starts the encounter with an envelope containing 1-2 puzzle pieces with a symptom or vital sign on it – when the enter the room they see a box in the middle of the room with “What diagnostic criteria does your patient have for pre-eclampsia?” | | |
| --- | --- | --- |
| 1. Blood pressure 165/85 2. Heart rate 108 3. Respiration rate 16 4. Oxygen saturation 98% 5. Fetal heart rate tracing: baseline 165, moderate variability, no accels, no decels 6. “I have a headache” 7. “I have heart palpitations” 8. “I have nausea” 9. “My legs are very swollen” 10. “I have a history of pre-eclampsia in my last pregnancy”     These are the clues that students start with – they put these two together to get the first code that unlocks the box below.   | If unable to identify adequate criteria, provide hint (What are some symptoms or labs that would diagnose someone with pre-eclampsia? Is that symptom truly diagnostic for pre-eclampsia?) | Learning objective 1  Learning objective 2 |
| Solution Step 1: Blood pressure and headache puzzle pieces connect and on the reverse is a code that opens the box | | |
| **Step 2:** Inside the box are many vials and bottles of medications along with a large envelope with a lock on it containing the next step. On the inside cover of the box it says “Which ones can be used to acutely treat severe range blood pressures?” There is also a phrase inside the box instructing students to enter medications in alphabetical order. | | |
| 1) IV Hydralazine 2) IV Labetalol 3) PO Nifedipine   Others that will also have numbers as red herrings    This is what the medications look like – they all have numbers on them | Students should identify that there are three different first line medications. If unable to identify the correct passcode, offer a targeted hint (Do you have the right medications? Are they in alphabetical order?) | Learning objective 3 |
| Solution Step 2: the correct medications each give a number and the students will use this to unlock the padded envelope | | |
| **Step 3:** Inside the envelope are several puzzle pieces and a black light. A note reads “Which labs are ordered in the evaluation of pre-eclampsia? (Including labs used to diagnose and as markers of severity of disease)” | | |
| 1) CBC 2) AST / ALT 3) Creatinine 4) LDH 5) Uric Acid 6) Urine protein / creatinine ratio   **Other puzzle pieces with other labs as red herrings**    This is what the large envelope looks like – it includes the puzzle pieces, the instructions, and a black light pen    This is what the puzzle looks like when assembled | If students cannot identify the appropriate labs, offer targeted hints (What other organ systems can be affected by pre-eclampsia? Are there any labs that can tell us about disease severity?) | Learning objective 2  Learning objective 4 |
| Solution Step 3: the 6 pieces with the correct labs will fit together and on the back of the puzzle says “Manage this seizure! (Hint: remember the whole patient and room)” | | |
| **Step 4:** The manikin in the room seizes! The students are supposed to identify things regarding the patient / room that they would do in the setting of a seizure. | | |
| Call for help! (on the emergency cord on the wall)  Put bed back together (on the foot of the bed that had been taken off to prep for delivery)  Handrails up (on the handrail that is down)  Put bed flat (on the button to lay the bed flat)  Turn patient on side (on the back of the patient)  Suction (on the wall cannister)  IV access (on patient forearm)  Supplemental O2 (on facemask)  Give magnesium sulfate (on the bag already hanging on the IV pole)    This shows the patient and bed with cards on the arm rail that is down, the IV, and the foot of the bed that has been pushed under the patient to allow for delivery  This shows two additional cards on the IV magnesium sulfate medication and the emergency call button    This is what the cards look like when the black light pen is shined on them, revealing clues | If students do not recognize the cards or the black light after a few minutes, facilitators can provide a hint. There is one card on the back of the patient; once the manikin is turned on her side that card is identified the seizure can stop. | Learning objective 5 |
| Solution Step 4: the cards have clues written in black light ink. The students should think to use the black light that was included in the last clue for these cards to read what is on the cards. | | |
| **Step 5:** the cards have clues to a crossword puzzle. Pens and blank crossword puzzles are in the room and the students should think that the answers to the questions on the cards fill in the blanks on the crossword puzzle. | | |
| The clues and answers are:   1- How long is magnesium usually continued after delivery? (twenty four hours) 2- What medication is given during pregnancy to help prevent development of pre-eclampsia? (baby aspirin) 3- What happens to maternal peripheral resistance in a normal pregnancy? (decreases) 4- What happens to maternal peripheral resistance in a pregnancy affected by pre-eclampsia? (increases) 5- What is the biggest risk factor for development of pre-eclampsia? (personal history) 6- This is a contraindication to the use of magnesium sulfate. (myasthenia gravis) 7- This medication can be given in cases of magnesium toxicity. (calcium gluconate) 8- What is one very important physical exam finding to check when a patient is on magnesium therapy? (reflexes) 9- What system processes magnesium for excretion from the body? (renal)     Blank crossword puzzles and passphrase sheets and pens are in the room    This slide is projected on a TV screen in the room so that the students can see that there is room for 9 orange cards (a clue that there are 9 things to look for in the room to manage the seizure) and how to decipher the final passphrase based on the results from the crossword. Once the preceptors hear the final passphrase, the slide is manually changed to the following.   | Students may use the correct clinical reasoning but enter incorrect crossword terms.  If the crossword is fully completed yet the final passphrase remains unsolved because of inaccurate wording, facilitators may give targeted hints to correct specific entries. | Learning objective 1  Learning objective 6 |
| Solution Step 5: Once completely filled out, the crossword has letters indicated that will spell out the final passphrase: We Love OB/GYN! | | |

Anticipated Mistakes

1. ***Escape room concept as uncommon in medical education: We found some students are so familiar with the simulation setting and repeatedly attempted to ask questions of the manikin or actually administer medications. A thorough review of what an escape room is and the expectations and instructions at the time of orientation to the experience is important.***
2. *Finding the cards with invisible ink prior to that step of the escape room: some teams may identify the cards intended to be discovered during the seizure earlier in the escape room. This is ok, but the students will eventually have to recognize that the blacklight is intended for those cards. Teams may require a hint to use the blacklight if the cards are noted early in the escape room.*
2. *Failure of team members to share clues they have found: Frequently, a learner will find a clue, or an aid to answer a clue, but will not communicate this out loud to the team, resulting in delays. This could be addressed in the debrief.*
3. *Difficulty identifying the appropriate code to move past Step 2: There are three first line medications which all have numbers on them. These numbers can be arranged in many combinations, but only one combination is the correct code. If students have the correct medication but are not getting the correct code, the facilitator may need to hint to try all combinations of the numbers until the lock opens.*
4. *Difficulty identifying steps to manage seizure: Some teams may see the manikin seizing but be unsure as to what is needed to move onto the next step of the escape room. If the students are not attempting to manage the seizure or engaging with their surroundings, the facilitator can prompt them by asking what they would do to manage a seizure. Facilitators can also provide targeted hints if certain cards are left undiscovered.*
5. *Failure to notice the blacklight: Learners may miss the blacklight in the Step 3 envelope or fail to link it to the cards from Step 4. Facilitators may prompt them by suggesting they re-check the envelope for items that could help them advance past Step 4.*
6. *Specific phrasing in the crossword: many groups struggled with the crossword phrasing of certain words trying “aspirin, baby aspirin, low dose aspirin” for example but we recognize that some of this struggle is part of a crossword puzzle itself. We also use the specific phrasing found in the escape room crossword in the initial didactic for continuity. As noted if groups struggle on any step for longer than 4 minutes we would provide a hint.*
