# Supplementary material for: The Eclampsia Escape Room: An Innovative Educational Experience for the OB/GYN Clerkship
Source: MedEdPORTAL. 2026 Aug 28;22:11629. doi: 10.15766/mep_2374-8265.11629 (PMC13522248; doi:10.15766/mep_2374-8265.11629)
Supplement: Supplementary file 1 [file mep_2374-8265.11629-s001.zip › D. Orientation for Students.docx]

**Appendix D:** This document serves as the student orientation and preparation. Facilitators will read and review the following information with participants. There are separate instructions for those participating in the optional high resource and low resource settings.

**Preparation and Orientation for Students**

Preparation and orientation for students – **high resource**:

Background: You are the intern on L&D days. This patient is a 40 yo G2P1001 at 40w5d who has just started pushing with the nurse. She wants you to come see her as she “just does not feel well”.

Instructions: This is NOT a simulation (i.e. you are not expected to talk with the manikin, examine the manikin, perform procedures or give medications). Instead, you should explore and interact with the environment in order to find clues and work together to solve a series of puzzles in order to figure out the final pass phrase. This will “win” the escape room!

Ground rules:

- Don’t force anything open if you do not yet have the correct clue or answer to open a box
- Don’t write on anything but paper
- Stay within the curtained area
- You can always leave the room if you need to (you are not locked in!)

-----------------------------------------------------------------------------------------------------------------------------

Preparation and orientation for students – **low resource**:

Background: You are the intern on L&D days. This patient is a 40 yo G2P1001 at 40w5d who has just started pushing with the nurse. She wants you to come see her as she “just does not feel well”.

Instructions: This is a series of puzzles that you will solve in order to find clues and work together to figure out the final pass phrase. This will “win” the escape room!

Ground rules:

- Don’t force anything open – you will receive permission from the facilitator to open envelopes once you have achieved the current puzzle step
- You will be provided with all materials to complete the puzzles, you do not need to explore the room
- You can always leave the room if you need to (you are not locked in!)
