# Supplementary material for: The Eclampsia Escape Room: An Innovative Educational Experience for the OB/GYN Clerkship
Source: MedEdPORTAL. 2026 Aug 28;22:11629. doi: 10.15766/mep_2374-8265.11629 (PMC13522248; doi:10.15766/mep_2374-8265.11629)
Supplement: Supplementary file 1 [file mep_2374-8265.11629-s001.zip › F. Debrief Tool.docx]

**Appendix F:** This document serves to help the facilitator debrief after the escape room encounter has been completed. We recommend starting the debrief with open ended questions and then proceeding with identifying each step and correlating with the associated learning objective. Areas that groups struggled with or incorrectly answered should be emphasized and reviewed.

**Eclampsia Escape Room Debrief Tool**

**Open ended questions:**

- How did this experience feel?
- What parts were challenging?
- What worked well? What parts could have gone better?

**Steps of Puzzle and Learning Objective Aligned Questions:**

**Step 1** was: *Understand the diagnostic criteria for pre-eclampsia and apply it to a specific patient to obtain the code to open the first box (high resource) or permission to open the large manila envelope (low resource)*

This corresponds to learning objective 2: Diagnose and distinguish gestational hypertension, pre-eclampsia, pre-eclampsia with severe features, chronic hypertension, and chronic hypertension with superimposed pre-eclampsia.

Debrief prompts:

- What diagnosis best fits this patient? Why?
- What features made this **severe**?
- How would this differ if she had chronic HTN?

High-yield distinctions:

- Gestational HTN: BP ≥140/90 after 20 wks, no proteinuria
- Pre-eclampsia: HTN + proteinuria **or** end-organ dysfunction
- Severe features: BP ≥160/110, CNS symptoms, LFTs ↑, Plt ↓, Cr ↑, pulm edema
- Chronic HTN: HTN before 20 wks
- Superimposed pre-eclampsia: new proteinuria/severe features in chronic HTN

**Step 2** was: *Recognize the need for urgent treatment of severe range blood pressure and use the correct medications in management to obtain the code to open the zipper pouch (high resource) or permission to open the small envelope (low resource)*

This corresponds to learning objective 3: Manage severe hypertension in pregnancy with appropriate medications and algorithms.

Debrief prompts:

- When do you treat BP urgently?
- Which meds are used? Is there an evidence-based best order?

High-yield algorithm:

- Treat ≥160 systolic or ≥110 diastolic or both
- First-line:
  - IV labetalol
  - IV hydralazine
  - PO nifedipine immediate release
- Goal: reduce stroke risk, not normalize BP

**Step 3** was: *Order appropriate labs to further evaluate for pre-eclampsia to complete the puzzle*

This corresponds to learning objective 4: Order and interpret labs for the evaluation of pre-eclampsia.

Debrief prompts:

- Which labs are used to evaluate?
- Which labs are diagnostic?
- What labs indicate severity?

High-yield labs:

- CBC → platelets
- CMP → AST/ALT, creatinine
- Urine protein (P/C ratio or 24-hr)
- Severe features = Plt <100k, Cr ≥1.1 or doubling, AST/ALT ≥2× ULN
- LDH can indicate hemolysis
- Uric acid can precede disease and as indicator of disease severity

**Step 4** was: *Manage a seizure including calling for help, patient positioning, airway management, magnesium sulfate administration, and other room and safety factors to find all crossword clues around the room (high resource) or be given all crossword clues (low resource)*

This corresponds to learning objective 5: Manage a seizure in pregnancy including use of magnesium sulfate and appropriate precautions.

Debrief prompts:

- First step after seizure?
- Room / bed considerations in this case?
- Magnesium dosing?
- Signs of magnesium toxicity?

High-yield management:

- Remember seizure precautions just like anywhere else in the hospital! Call for help, put bed rails up, put the bed back together if broken down for delivery, lay head of bed flat, turn the patient on their side, provide suction, provide oxygen, ensure IV access, give magnesium sulfate for eclampsia
- MgSO₄ loading: 4–6 g IV
- Maintenance: 1–2 g/hr
- Magnesium does not treat the current treatment; it prevents seizure recurrence
- Toxicity: ↓ reflexes, resp depression
- Antidote: calcium gluconate
- Airway protection emphasized

**Step 5** was: *Apply knowledge about physiology and other considerations for pre-eclampsia management to complete the final crossword*

This corresponds to learning objective 1: Describe how normal pregnancy physiology changes and how the pathophysiology of pre-eclampsia differs.

Debrief prompts:

- What normal cardiovascular changes occur in pregnancy?
- What pathophysiologic process drives pre-eclampsia?

High-yield teaching points:

- Normal: ↓ SVR, ↑ plasma volume, ↓ BP mid-pregnancy
- Pre-eclampsia: abnormal placentation → endothelial dysfunction → ↑SVR, end-organ injury

Step 5 also corresponds to learning objective 6: Discuss potential sequelae of pre-eclampsia and future considerations for prevention.

Debrief prompts:

- Maternal and fetal complications?
- Long-term risks?
- Prevention strategies?

High-yield sequelae:

- Maternal: stroke, HELLP, renal disease, eclampsia
- Fetal: growth restriction, preterm birth
- Prevention: low-dose aspirin in high-risk patients, postpartum BP follow-up
