# Supplementary material for: The Eclampsia Escape Room: An Innovative Educational Experience for the OB/GYN Clerkship
Source: MedEdPORTAL. 2026 Aug 28;22:11629. doi: 10.15766/mep_2374-8265.11629 (PMC13522248; doi:10.15766/mep_2374-8265.11629)
Supplement: Supplementary file 1 [file mep_2374-8265.11629-s001.zip › G. Feedback Survey.docx]

**Appendix G:** This survey was given to all participants to gather information on their satisfaction and perceived value of the escape room as well as to assess whether students feel learning objectives were met. We also evaluated for things that went well and things that could be improved with the experience. This is an optional component for ongoing evaluation if the escape room is implemented at other institutions.

**Eclampsia Escape Room Survey**

1. How satisfied were you after participating in the Eclampsia Escape Room as a component of the overall educational experience today?

1 – Very satisfied

2 – Satisfied

3 – Neutral

4 – Unsatisfied

5 – Very unsatisfied

2. How much did you feel that participation in the Eclampsia Escape Room added to the value of the overall educational experience today?

1 – Significant value added

2 – Value added

3 – Neutral

4 – Detraction

5 – Significant detraction

3. Did you think that the Eclampsia Escape Room met the proposed learning objectives? Why?

1 – Yes

2 – Somewhat

3 – No

Why? __________________________________________________________________________________________________________________________________________________________________________________________________________________________________________

4. What worked well or was satisfying in the Eclampsia Escape Room?
5. What did not work well or was frustrating in the Eclampsia Escape Room?
6. Comments or suggestions to improve:
